# Metabolic syndrome and early stage breast cancer outcome: results from a prospective observational study

**DOI:** 10.1007/s10549-020-05701-7

**Published:** 2020-06-04

**Authors:** Giuseppe Buono, Anna Crispo, Mario Giuliano, Carmine De Angelis, Francesco Schettini, Valeria Forestieri, Rossella Lauria, Michelino De Laurentiis, Pietro De Placido, Carmen Giusy Rea, Carmen Pacilio, Emanuela Esposito, Maria Grimaldi, Flavia Nocerino, Giuseppe Porciello, Aldo Giudice, Alfonso Amore, Anita Minopoli, Gerardo Botti, Sabino De Placido, Meghana V. Trivedi, Grazia Arpino

**Affiliations:** 1 Department of Clinical Medicine and Surgery, Oncology Division, University of Naples Federico II, Naples, Italy; 2 Epidemiology and Biostatistics Unit, Istituto Nazionale Tumori - IRCCS - Fondazione G. Pascale, Naples, Italy; 3 Lester and Sue Smith Breast Center, Baylor College of Medicine, Houston, TX USA; 4 Breast Unit, Istituto Nazionale Tumori - IRCCS - Fondazione G. Pascale, Naples, Italy; 5 Laboratory Medicine Unit, Istituto Nazionale Tumori - IRCCS - Fondazione G. Pascale, Naples, Italy; 6 Scientific Direction, Istituto Nazionale Tumori - IRCCS - Fondazione G. Pascale, Naples, Italy; 7 Departments of Pharmacy Practice and Translational Research and of Pharmacological and Pharmaceutical Sciences, College of Pharmacy, University of Houston, Houston, TX USA

**Keywords:** Breast cancer, Metabolic syndrome, Metabolic syndrome components, Breast cancer outcome

## Abstract

**Purpose:**

Obesity and insulin resistance have been associated with poor prognosis in breast cancer (BC). The present prospective study aimed to investigate the impact of metabolic syndrome (MetS) and its components on early BC (eBC) patients’ outcome.

**Methods:**

MetS was defined by the presence of 3 to 5 of the following components: waist circumference > 88 cm, blood pressure ≥ 130/≥ 85 mmHg, serum levels of triglycerides ≥ 150 mg/dL, high density lipoprotein < 50 mg/dL and fasting glucose ≥ 110 mg/dL. Seven hundred and seventeen patients with data on ≥ 4 MetS components at BC diagnosis were enrolled. Study population was divided into two groups: patients with < 3 (non-MetS) vs. ≥ 3 components (MetS). Categorical variables were analyzed by Chi-square test and survival data by log-rank test and Cox proportional hazards regression model**.**

**Results:**

Overall, 544 (75.9%) and 173 (24.1%) women were categorized as non-MetS and MetS, respectively. MetS patients were more likely to be older, postmenopausal, and insulin-resistant compared to non-MetS patients (*p* < 0.05). In multivariate analysis, MetS patients had a numerically higher risk of relapse [disease-free survival (DFS), hazard ratio (HR) 1.51, *p* = 0.07] and a significantly higher risk of death compared to non-MetS patients [overall survival (OS), HR 3.01, *p* < 0.0001; breast cancer-specific survival (BCSS), HR 3.16, *p* = 0.001]. Additionally, patients with 1 to 2 components of MetS had an increased risk of dying compared to patients with 0 components (OS, HR 4.90, *p* = 0.01; BCSS, HR 6.07, *p* = 0.02).

**Conclusions:**

MetS correlated with poor outcome in eBC patients. Among patients without full criteria for MetS diagnosis, the presence of 1 or 2 components of the syndrome may predict for worse survival.

## Introduction

Breast cancer (BC) represents the most common cancer among women, with about 2 million of new cancer cases estimated in 2018 worldwide. Incidence rate varies across world regions, ranging from 26 to 28 per 100,000 in developing countries (i.e., South-Central Asia and Middle Africa) to 92–94 per 100,000 in the more developed ones (i.e., Western Europe, Australia, and New Zealand) [1]. This difference in BC incidence can be explained by different dietary and nutritional habits with a higher consumption of fatty, low-fiber, and processed food in westernized countries [2, 3]. This unhealthy diet, often correlated with physical inactivity, is considered one of the most important causes of the so-called “obesity epidemic” [4], associated with cardiovascular events and deaths [5].

Obesity has been associated with postmenopausal BC incidence [6, 7], BC subtypes [8] and poor survival [9] through different mechanisms including an increased estrogen production from circulating androgens [10] and the promotion of a low-grade chronic inflammation state [11, 12]. Similarly, diabetes has been correlated with increased BC risk [13] and poor survival [14, 15], in part due to the activation of the oncogenic Ras-MAPK and PI3K/Akt pathways in breast cells [16, 17].

Abdominal obesity and high fasting glycaemia combined with dyslipidemia and hypertension are diagnostic criteria of a more complex metabolic disorder known as “Metabolic Syndrome” (MetS) [18]. Initially linked to cardiovascular diseases, MetS has been recently associated with increased cancer risk [19], particularly colon-rectal [20] and BC [21, 22] in previous studies.

We have previously demonstrated that the concomitant presence of obesity and diabetes worsened early breast cancer (eBC) patients’ outcome compared to presence of only one or none of these comorbidities [23], suggesting not only a close link between these medical conditions, but also an outcome worsening as the number of the metabolic alterations increased.

We therefore hypothesized that MetS could be associated with a worse outcome in eBC patients. Large studies [24–26] retrospectively correlated MetS with worse prognosis in eBC patients. However, prospective studies evaluating the association between MetS and eBC patients’ long-term outcome are still missing. In this study, we comprehensively investigated the association between MetS or its individual components and BC outcome in a large prospective cohort of eBC patients.

## Materials and methods

### Study population

Overall, 955 eBC patients were prospectively enrolled in this study between January 2009 and December 2013 at University Hospital Federico II and National Cancer Institute G. Pascale, Naples, Italy. Clinical data [age, height, weight, waist circumference, blood pressure, fasting glucose, triglycerides, total cholesterol, high density lipoprotein (HDL), low density lipoprotein (LDL)] and tumor characteristics [tumor size (T), nodal status (N), tumor stage, estrogen receptor (ER) and progesterone receptor (PgR) expression, grading (G), ki67, HER2 status] were collected before starting systemic (neo)adjuvant therapy. The homeostatic model assessment for insulin resistance (HOMA-IR) score was calculated as fasting glucose (mmol/l) multiplied by fasting insulin (µUI/l) divided by 22.5 [26]. Immunohistochemical (IHC) BC subtypes were identified and categorized according to the 13th St. Gallen International Breast Cancer Conference (2013) Expert Panel [27].

**Fig. 1:**
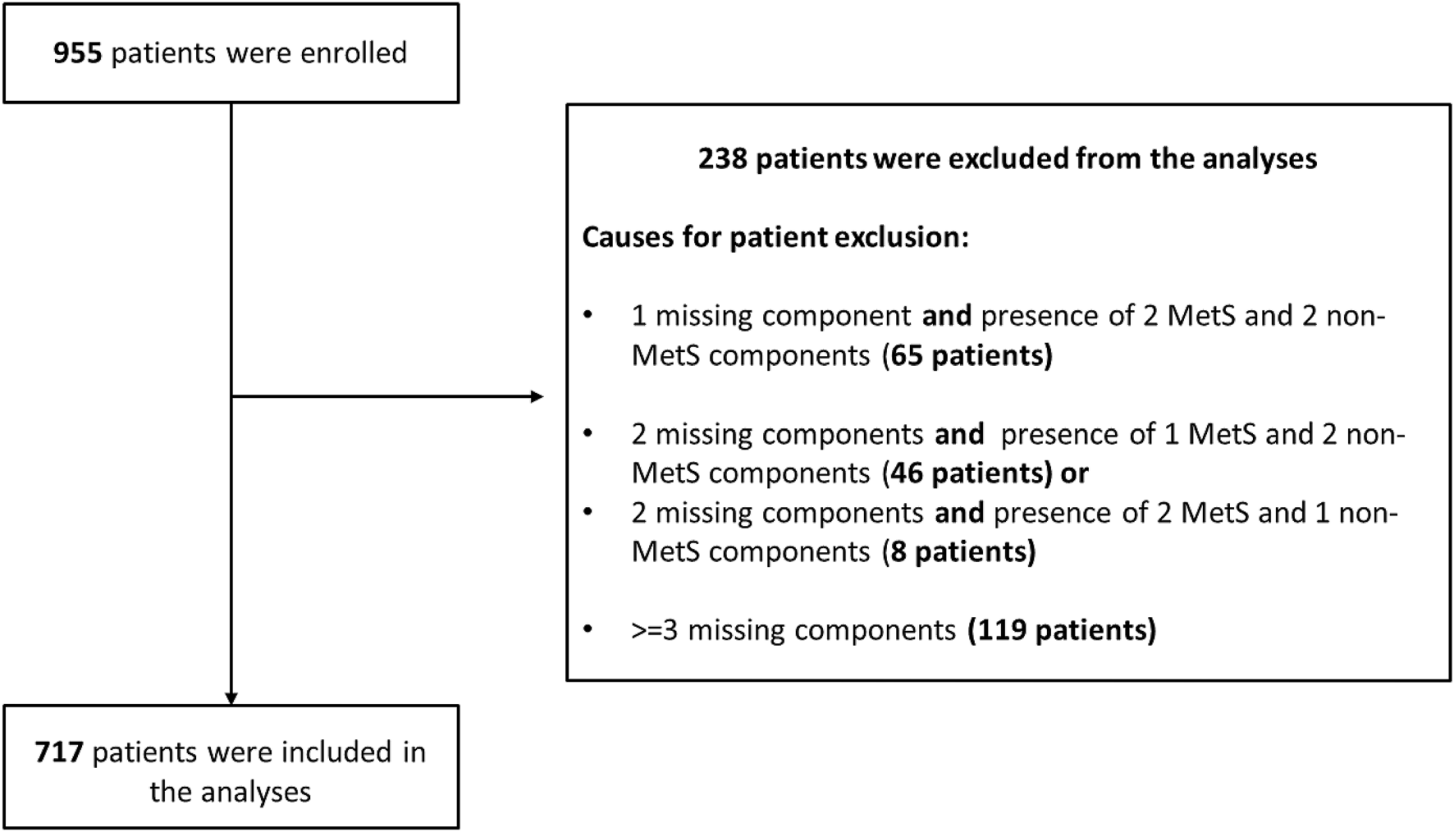
Study flow chart. Metabolic Syndrome (MetS) was defined by the presence of 3 of the following variables: waist circumference > 88 cm, blood pressure ≥ 130/ ≥ 85 mmHg, triglycerides ≥ 150 mg/dL, HDL < 50 mg/dL and fasting glucose ≥ 110 mg/dL. Patients were excluded from the study if they had missing components that precluded investigators from assessing accurate MetS status as detailed in the flow chart

MetS was defined by the presence of 3 to 5 of the following variables: waist circumference > 88 cm, blood pressure ≥ 130/ ≥ 85 mmHg, triglycerides ≥ 150 mg/dL, HDL < 50 mg/dL and fasting glucose ≥ 110 mg/dL, according to the National Cholesterol Education Program Expert Panel on Detection, Evaluation, And Treatment of High Blood Cholesterol In Adults—NCEP-ATPIII criteria [28]. A total of 717 patients (75.1%) had complete data to define or not the presence of MetS, thus were included in the current analysis (Fig. 1). Study population was divided into 2 main groups: (1) patients with less than 3 components (non-MetS); (2) patients with 3 or more components (MetS). The study was approved by the Institutional Review Board of the University of Naples Federico II (IRB approval number 75/15) and participants provided written informed consent to participate. The records and data of patients were anonymized and de-identified prior to analysis.

### Statistical analyses

Descriptive statistics for the categorical data were reported. The Chi-square test was used to assess the association between MetS and non-MetS groups and clinico-pathological variables. Patients’ outcomes were analyzed in terms of disease-free survival (DFS; with local, contralateral, and distant disease recurrence as well as secondary primary tumors and death from any cause defined as the event), overall survival (OS; with death from any cause defined as the event) and breast cancer-specific survival (BCSS; death from the disease). Univariate analyses were performed using the Kaplan–Meier method. Numbers of events and survival percentage were reported, and Log-rank test was conducted to determine the statistically significant difference. Cox multivariate analysis for DFS, OS and BCSS was done either for singular components of MetS or for the combination of them into “number of MetS components”. For each variable, the best category was used as the reference group for the calculations of hazard ratios (HR) and 95% confidence intervals (CI). Statistical analyses were performed using IBM® SPSS® Statistics, version 25 (IBM Corp., Armonk, NY, USA). All statistical analyses were of an exploratory nature, with p values of less than 0.05 considered significant, without any adjustments for multiplicity applied.

## Results

### Patient demographics, clinical and pathological characteristics

Overall, 173 (24.1%) and 544 (75.9%) women were categorized as MetS and non-MetS, respectively. Clinical data and tumor characteristics, according to the presence or absence of MetS, are reported in Table 1. MetS group had more elderly [age > 55 years; 130 (75.1%) vs. 198 (36.4%), *p* < 0.0001] and postmenopausal [152 (87.9%) vs. 271 (49.8%), *p* < 0.0001] women than non-MetS groups**.** Patients with MetS were also more likely to be insulin-resistant, as HOMA-IR score higher than 5 was found in 43 out of 173 (29.3%) MetS vs. 25 out of 544 (5.7%) non-MetS groups (*p* < 0.0001).

**Table 1:** Distribution of patients’ clinico-pathological and metabolic characteristics

Characteristics	non-MetS 544 (75.9%)	MetS 173 (24.1%)	*p*-value
Age			< 0.0001
≤ 55 years	346 (63.6)	43 (24.9)	
> 55 years	198 (36.4)	130 (75.1)	
Menopause			< 0.0001
Post-menopause	271 (49.8)	152 (87.9)	
Pre-menopause	273 (50.2)	21 (12.1)	
Stage			0.2
I–II	433 (82.6)	127 (78.4)	
III	91 (17.4)	35 (21.6)	
Therapy			0.4
Chemo only	80 (15.4)	25 (15.9)	
Hormone only	188 (36.2)	65 (41.4)	
Chemo + hormone	251 (48.4)	67 (42.7)	
IHC-subtypes^a^			0.7
Luminal A	207 (39.7)	71 (42.0)	
Luminal B	149 (28.5)	48 (28.4)	
Her2 positive	91 (17.4)	31 (18.3)	
Triple negative	75 (14.4)	19 (11.2)	
HOMA-IR score			< 0.0001
Normal (< 2.6)	312 (71.7)	57 (38.8)	
Medium (2.6–5)	98 (22.5)	47 (32.0)	
High (> 5)	25 (5.7)	43 (29.3)	
Patient status			< 0.0001
Alive without disease relapse	385 (70.8)	97 (56.1)	
Alive with disease relapse	68 (12.5)	15 (8.7)	
Death for the disease	41 (7.5)	34 (19.7)	
Death for other causes	7 (1.3)	7 (4.0)	
Lost to follow-up	43 (7.9)	20 (11.6)	

*IHC* immunohistochemical

^a^St Gallen categorization 2013: Luminal A: ER+, PgR >  = 20%, ki67 < 20%, Her2−; Luminal B: ER+; PgR < 20% or ki67 >  = 20%; Her2−; Her2positive: any ER and PgR, any ki67, Her2+; Triple negative: ER−; PgR−; Her2−; ki67 any

^b^NCEP—ATP III criteria

Death rates for BC and other causes were higher in patients with MetS vs non-MetS. Incidence for death for BC and death for other causes were 34 (19.7%) vs. 41 (7.5%) and 7 (4.0%) vs. 7 (1.3%) in patients with MetS vs non-MetS, respectively (*p* < 0.0001).

No statistically significant differences in tumor stage or IHC-subtypes were identified between the two groups and the presence of MetS did not influence the choice of (neo)adjuvant systemic therapy.

### Survival analysis

**Fig. 2:**
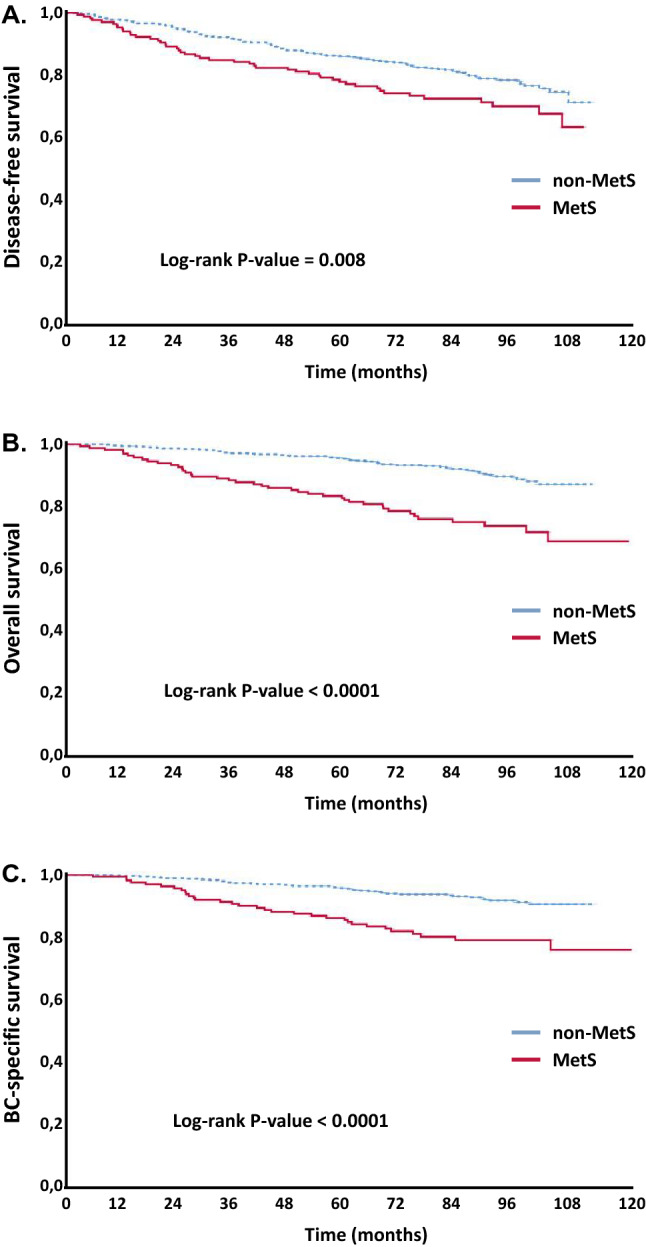
Disease-free Survival (**a**), Overall Survival (**b**) and Breast Cancer-Specific Survival (**c**) according to metabolic syndrome (MetS)

**Fig. 3:**
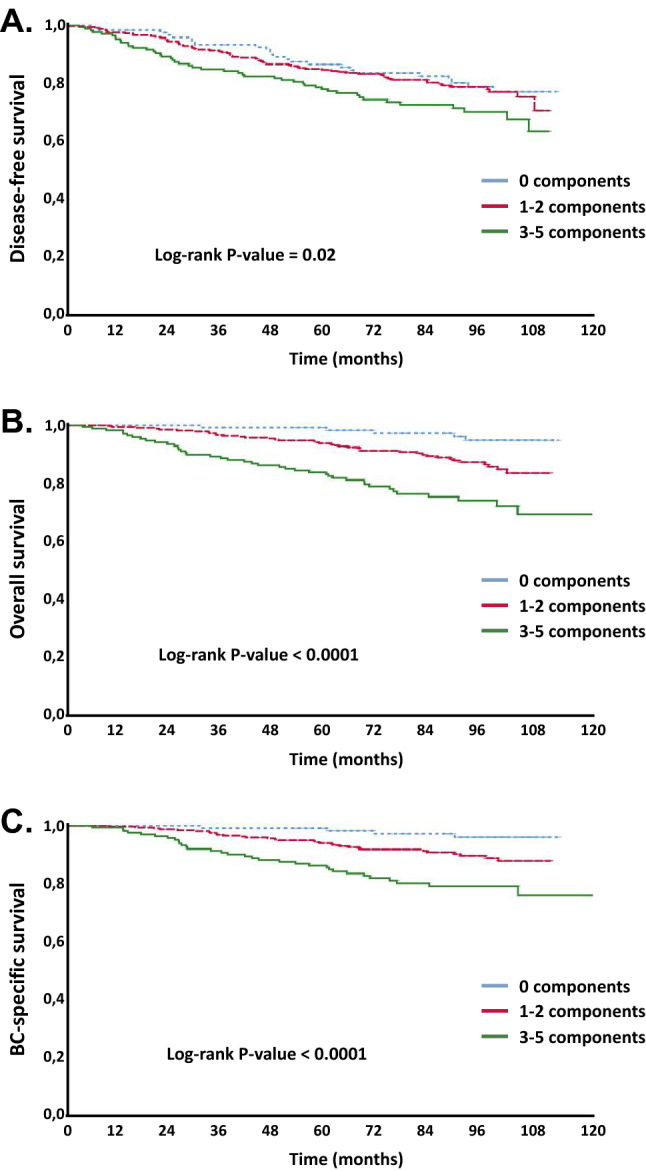
Disease-free Survival (**a**), Overall Survival (**b**) and Breast Cancer-Specific Survival (**c**) according to number of MetS components

In univariate analysis, patients with MetS were more likely to recur and die from BC. After a median follow-up time of 7.1 years from diagnosis, rates for DFS, OS and BCSS were 71.2% vs. 79.8% (*p* = 0.008), 75.9% vs. 91.1% (*p* < 0.0001) and 80.0% vs. 92.4% (*p* < 0.0001), in patients with MetS vs. non-MetS, respectively (Fig. 2a–c; Table 2). Interestingly, among patients with non-MetS, patients with 1 or 2 components of MetS had an increased risk of recurrence and death for BC compared to patients with no component at all. However, these risks were lower when compared with those of patients with MetS. Specifically, rates for DFS, OS and BCSS were 80.2% vs. 79.2% vs. 71.2% (*p* = 0.02), 98.9% vs. 88.1% vs. 75.9% (*p* < 0.0001) and 96.7% vs. 89.9% vs. 80.0% (*p* < 0.0001), in patients with ≥ 3 vs. 1–2 vs. 0 components of MetS (Fig. 3a–c; Table 2). Other factors associated to both DFS and OS rates in univariate analysis were tumor stage, IHC-subtypes, type of (neo)adjuvant therapy, triglycerides, and fasting glucose levels. Age, waist circumference, blood pressure and HDL levels correlated to OS only (Table 2).

**Table 2:** Disease-free survival and overall survival results: Univariate analysis

Variable	DFS rates			OS rates		
	No. of events	*%*	Log-rank^a^*p*-value	No. of events	*%*	Log-rank^a^*p*-value
MetS			0.008			< 0.0001
No	109	79.8		48	91.1	
Yes	49	71.2		41	75.9	
No. of MetS comp.^b^			0.02			< 0.0001
0 components	24	80.2		5	98.9	
1–2 components	68	79.2		39	88.1	
3–5 components	49	71.2		41	75.9	
Age			0.1			< 0.0001
≤ 55 years	78	79.8		26	93.3	
> 55 years	80	75.2		63	80.5	
Stage			< 0.0001			< 0.0001
I–II	100	82.1		456	90.0	
III	46	62.0		30	75.2	
IHC-subtypes			0.001			0.04
Luminal A	52	81.2		32	88.4	
Luminal B	41	79		24	87.7	
HER2 positive	36	70		16	86.7	
Triple negative	25	72.8		16	82.6	
Therapy			< 0.0001			< 0.0001
Chemotherapy only	32	69.5		22	79.0	
Hormone only	34	86.6		22	91.3	
Chemo + hormone	70	78.0		29	90.9	
HOMA-IR score			0.9			0.1
Normal (< 2.6)	83	77.3		51	86.1	
Medium (2.6–5)	26	81.9		10	93.1	
High (> 5)	19	71.2		8	87.9	
Waist circumference			0.1			< 0.0001
≤ 88 cm	72	79.8		30	91.6	
> 88 cm	84	75.5		58	83.1	
Blood pressure			0.8			0.03
< 130; < 85 mmHg	80	78.1		40	89.0	
≥ 130; ≥ 85 mmHg	64	77.5		47	83.5	
HDL			0.1			0.04
≥ 50 mg/dL	93	77.6		52	87.5	
< 50 mg/dL	37	72.6		26	80.7	
Triglycerides			< 0.0001			< 0.0001
< 150 mg/dL	110	80.8		44	92.3	
≥ 150 mg/dL	45	62.8		43	64.2	
Fasting glucose			0.003			0.001
< 110 mg/dL	123	79.2		64	89.2	
≥ 110 mg/dL	34	70.2		25	78.1	

^a^Kaplan–Meier univariate analysis

^b^NCEP—ATP III criteria

In Cox regression models (Table 3), adjusted for age, tumor stage, IHC-subtypes and therapy, MetS was associated with a threefold increased risk of BC mortality (HR for BCSS = 3.16, 95% confidence interval (CI) 1.64–6.07, *p* = 0.001) and of death (HR for OS = 3.01, 95% CI 1.72–5.28, *p* < 0.0001) compared to patients with non-MetS. A numerical, but not statistically significant, difference in DFS was observed. High waist circumference (> 88 cm), high blood pressure (≥ 130; ≥ 85 mmHg), high triglycerides (≥ 150 mg/dL), and high fasting glucose (≥ 110 mg/dL) were individually associated with increased risk of death (HR for OS = 2.34, 95% CI 1.32–4.14, *p* = 0.003; HR 1.99, 95% CI 1.15–3.64, *p* = 0.01; HR 3.58, 95% CI 2.08–6.17, *p* < 0.0001; and HR 2.26, 95% CI 1.26–4.05, *p* = 0.006, respectively) and death for BC (HR for BCSS = 3.24, 95% CI 1.64–6.41, *p* = 0.001; HR 2.02, 95% CI 1.07–3.81, *p* = 0.03; HR 3.10, 95% CI 1.59–6.05, *p* = 0.001; and HR 2.49, 95% CI 1.25–4.96, *p* = 0.009, respectively) in multivariable adjusted models (Table 3). For DFS, patients with triglycerides ≥ 150 mg/dL and fasting glucose ≥ 110 mg/dL had an increased risk of relapse (HR for DFS = 1.66, 95% CI 1.01–2.74, *p* = 0.04 and HR 1.70, 95% CI 1.04–2.76, *p* = 0.03, respectively).

**Table 3:** Cox multivariate analysis of BC risk for DFS, OS and BC-specific survival

Variable	Disease-free survival			Overall-survival			BC-specific survival		
	HR	(95% CI)	*p*-value	HR	(95% CI)	p-value	HR	(95% CI)	*p*-value
MetS									
No	1^†^			1^†^			1^†^		
Yes	1.51	0.96–2.38	0.07	3.01	1.72–5.28	< 0.0001	3.16	1.64–6.07	0.001
Waist circumference									
≤ 88 cm	1^†^			1^†^			1^†^		
> 88 cm	1.36	0.91–2.02	0.1	2.34	1.32–4.14	0.003	3.24	1.64–6.41	0.001
Blood pressure									
< 130; < 85 mmHg	1^†^			1^†^			1^†^		
≥ 130; ≥ 85 mmHg	1.26	0.83–1.92	0.3	1.99	1.15–3.64	0.01	2.02	1.07–3.81	0.03
HDL									
≥ 50 mg/dL	1^†^			1^†^			1^†^		
< 50 mg/dL	0.86	0.53–1.38	0.5	0.65	0.35–1.17	0.1	0.54	0.27–1.05	0.07
Triglycerides									
< 150 mg/dL	1^†^			1^†^			1^†^		
≥ 150 mg/dL	1.66	1.01–2.74	0.04	3.58	2.08–6.17	< 0.0001	3.10	1.59–6.05	0.001
Fasting glucose									
< 110 mg/dL	1^†^			1^†^			1^†^		
≥ 110 mg/dL	1.70	1.04–2.76	0.03	2.26	1.26–4.05	0.006	2.49	1.25–4.96	0.009
Number of MetS comp.^a^			0.04			0.001			0.003
0 components	1^†^			1^†^			1^†^		
1–2 components	1.48	0.86–2.57	0.1	4.90	1.47–16.35	0.01	6.07	1.41–26.21	0.02
3–5 components	2.26	1.18–4.33	0.01	12.2	3.49–43.01	< 0.0001	15.97	3.49–73.16	< 0.0001

Adjusted for terms of Age (< 40,40–45,46–55,56–65,66–75,75 +); Stage (I–II, III); IHC- subtypes (Luminal A: ER+, PgR >  = 20%, ki67 < 20%, Her2−; Luminal B: E+;PgR < 20% or ki67 >  = 20%; Her2−; Her2positive: any ER and PgR, any ki67, Her2+; Triple negative: ER−; PgR−; Her2−; ki67 any) and Therapy (Chemotherapy only, Hormone only, Chemo + hormone)

^a^Wald test

Compared to individuals without any component of MetS present, the risk of death and death for BC increased steeply as the number of MetS components increased (Table 3). Patients with 3–5 components had over twofold higher risk of relapse (HR for DFS = 2.26, 95% CI 1.18–4.33, *p* = 0.01), 12-fold higher risk of death (HR for OS = 12.2, 95% CI 3.49–43.01, *p* < 0.0001) and nearly 16-fold higher risk of death for BC (HR for BCSS = 15.97, 95% CI 3.49–73.16, *p* < 0.0001) than patients with 0 components (Table 3). Interestingly, patients with 1–2 MetS components presented about fivefold higher risk of death (HR for OS = 4.90, 95% CI 1.47–16.35, *p* = 0.01), sixfold risk of death for BC (HR for BCSS = 6.07, 95% CI 1.41–26.21, *p* = 0.02) but no significant increased risk of relapse compared to patients with no components at all (Table 3).

## Discussion

In this large prospective study, we have found that MetS was significantly associated with increased risk of dying in general and of dying from breast cancer in eBC patients receiving (neo)adjuvant therapy at a median follow-up time of 7.1 years. To our knowledge, this is one of the first prospective studies correlating MetS with poor long-term outcome in a large cohort of eBC patients in this setting.

Our findings are consistent with those from previous studies [24, 25, 29, 30]. In a cohort of 4,216 eBC patients, the presence of MetS at diagnosis was associated with a 1.5-fold increased risk of recurrence or second primary BC and 1.65-fold increased risk of BC-specific mortality compared with patients with no MetS [24]. Similarly, a study in 10,014 patients reported twofold increase in BC mortality with MetS [30], while another study (*N* = 288,834) reported a 23% higher risk of BC mortality in only older (> 60 years) women with MetS without any impact in younger patients [25]. Interestingly, MetS also correlated with an enhanced risk of new BC events (defined as loco-regional recurrences, distant metastasis or new primary BC) in a prospective study using 2,092 eBC patients; however, an impact on survival was not evaluated [29]. As other reports [29, 30], our study found that patients with MetS were more likely to be older and postmenopausal compared to those with no MetS. However, differently from previous reports [31, 32] MetS was not associated with adverse pathological features in our study, as no correlation between IHC-defined BC subtypes, tumor stage at diagnoses and presence of MetS was found. The presence of MetS at diagnoses also did not influence the choice of (neo)adjuvant treatment administered.

In our study, even the presence of a single component of MetS such as high waist circumference, blood pressure, fasting glucose or triglycerides, was strongly associated with increased risk of BC mortality, regardless other well-known prognostic factors such as age, tumor stage, IHC-subtypes and therapy. Other studies have also investigated the impact of individual MetS components on BC outcome with differing results. Higher risk of BC mortality was reported in women in the highest tertile of total cholesterol (29% higher risk) and blood pressure (41% higher risk) [33] and in patients with high waist circumference (HR 1.32), high cholesterol (HR 1.24), and hypertension (HR 1.24) [34]. In addition, BC outcomes correlated with hyperglycemia [35] and higher waist-to-hip ratio [36]. Increased insulin levels, due to insulin resistance (IR) also directly correlated with BC relapse and mortality [37]. However, we could not detect any significant correlation between HOMA-IR score and increased risk of BC relapse or mortality, which is consistently with previous findings from our group as well as others [23, 29]. These data suggest that a complex interaction between metabolic alterations caused by altered glucose metabolism, rather than the presence of IR alone, may be more relevant for BC outcome.

Interestingly, in this study, we also demonstrate that, among patients without MetS, the risk of BC mortality increased significantly as the number of MetS components increased. We observed a sixfold and 16-fold increase in risk of BC mortality among women with 1–2 components and 3–5 of MetS, respectively, compared with women with no components. These results indicate that higher the extent of metabolic health impairment, worse the outcomes in eBC patients.

The mechanisms by which MetS can increase BC risk and worsen patients’ prognosis are partially understood. Each of the metabolic alterations included in MetS may play a critical role in BC biology. Increased glycaemia and IR have shown to promote malignant cell growth [38]. Insulin mediates insulin like growth factor (IGF-1) production, resulting in a hyper-activation of Ras-MAPK and PI3K/Akt pathways in malignant cells [16] and increases serum free estrogen levels by reducing the concentration of the sex hormone binding globulin [39]. Obesity, not only promotes estrogen production, as the aromatase enzyme synthesizes estrogens in adipose tissue from circulating androgens [10], but is also associated with a low-grade chronic inflammation. This is characterized by reduced levels of anti-inflammatory cytokines (such as adiponectin) [11] and high levels of pro-inflammatory cytokines [12] [as tumor necrosis factor alfa (TNFα), interleukin (IL) 1β, IL-6 and IL-8] that can exert mitogenic, anti-apoptotic, and angiogenic effects, thus promoting disease progression. Importantly, in mammary gland, the interaction with BC cells may promote transformation of mammary adipocytes into the so-called “cancer associated adipocytes” (CAA), which may enhance tumor growth and progression [40] through lipolytic activity and the secretion of adipokines [41]. Moreover, the adipocyte/tumor cell crosstalk may negatively affect response to systemic treatment [42] and mediate endocrine resistance in BC cells, particularly in the presence of high glucose levels [43]. Pre-clinical studies have shown that dyslipidemia, in an Apolipoprotein (Apo) E knockout (ApoE^−/−^) mice model, promote tumor growth and metastasis development through activation of PI3K/Akt signal pathway [44] due to increased cholesterol levels. Finally, low serum HDL, as markers of increased androgen levels, are also associated with BC risk [45]. Taken together, multiple molecular mechanisms related to various metabolic alterations within MetS may be responsible for an increased risk of BC development and progression. These mechanisms may function independently in presence of only one metabolic alteration to impact patient outcome or cooperatively when multiple MetS components are present to further worsen the recurrence risk and survival.

Our study and findings have several strengths and limitations. First, our large prospective cohort of patients is fully characterized with regard to clinical and tumor features, objective baseline measures of MetS, and subsequent treatment. This information has allowed us to comprehensively evaluate the effect of MetS components on patients’ outcome by a multivariate model adjusted for known prognostic variables. Second, exposure and covariate data were obtained at baseline before treatment could interfere with the metabolic parameters included in the study. Third, data on BC mortality were obtained from patient’s charts, thereby minimizing the risk of death misclassification. On the other hand, we did not have information about the medical treatments for diabetes, hypertension, and dyslipidemia, which may have led to un underestimation of the number of patients with MetS. However, our analysis focused on the real-time laboratory results of patients may be more appropriate to assess functional/uncontrolled MetS. Future studies determining the effects of lifestyle and/or therapeutic interventions to treat MetS on BC progression, risk of recurrence, and survival may help improve clinical management of BC patients with MetS. In addition, small number of patients included in some of our sub-group analysis may have limited the power to detect significant associations between clinical variables and should be confirmed in future studies.

In summary, we demonstrate here that the presence of MetS at diagnosis correlates with poor outcome in eBC patients. Compared to patients without any criterion for MetS at diagnosis, patients with only 1 or 2 components of MetS have worse survival. In addition, the prognosis worsens with the presence of even more components of MetS. These findings strongly support testing for components of MetS in all eBC patients at diagnoses and during (neo)adjuvant treatment to improve survival.
